# The risk of complications in elective orthopedic surgeries in children and young adults with cerebral palsy: a population-based register study

**DOI:** 10.2340/17453674.2025.43705

**Published:** 2025-05-27

**Authors:** Anna TELLÉUS, Johan VON HEIDEKEN, Fredrik GRANATH, Eva BROSTRÖM, Gunnar HÄGGLUND, Per ÅSTRAND

**Affiliations:** 1 Department of Women’s and Children’s Health, Karolinska Institutet, Karolinska University Hospital, Stockholm; 2 Highly specialized pediatric orthopedics and pediatric medicine, Astrid Lindgren’s Children Hospital, Karolinska University Hospital, Stockholm; 3 Department of Medicine Solna, Division of Clinical Epidemiology, Karolinska Institutet, Stockholm; 4 Department of Clinical Sciences, Orthopedics, Lund University, Lund, Sweden

## Abstract

**Background and purpose:**

Musculoskeletal deformities in cerebral palsy (CP) may be surgically treated, but population-based studies of postoperative complications after these surgeries are rare. The aim of our study was to assess the risk of complications following elective orthopedic surgery in children and young adults with CP.

**Methods:**

We performed a register-based cohort study of 1,514 individuals born between 1990 and 2019 who underwent 2,983 orthopedic surgical events between January 1, 1997, and December 31, 2019. Data was obtained from the CP surveillance program CPUP, the Swedish National Patient Register, and the National Cause of Death Register. We used logistic regression to calculate odds ratios (OR) with 95% confidence intervals (CI) for postoperative complications within 90 days, in relation to the Gross Motor Function Classification System (GMFCS) level, anatomic level, and type of surgery (i.e., skeletal vs. soft tissue).

**Results:**

13% of all surgical events had at least 1 postoperative complication (6% in soft tissue surgeries, 17% in skeletal surgeries), and 51% of these were related to infection. The complication rate was higher in individuals with GMFCS levels IV and V than in the pooled GMFCS levels I–III. The highest ORs were found in GMFCS level V (7.0, CI 3.7–13.5) vs. GMFCS I and spinal surgery (7.9, CI 3.7–13.5) vs. foot/ankle surgery. The OR for skeletal surgery was 1.6 (CI 1.2–2.1) compared with soft tissue surgery.

**Conclusion:**

13% of all surgical events had at least 1 postoperative complication. The risk of complications after elective orthopedic surgery was higher in children with higher GMFCS levels and in skeletal surgery compared with soft tissue surgery.

Cerebral palsy (CP) is the most common neurological disorder causing motor impairment in children, affecting about 2 children per 1,000 in Sweden [1-3]. CP covers a broad range of clinical presentations. Impairments in gross motor function are usually classified into 5 levels using the Gross Motor Function Classification System (GMFCS). Children in GMFCS level I walk independently without any aids, whereas children in GMFCS levels IV and V use a wheelchair for transportation [4].

While musculoskeletal deformities secondary to altered brain function in CP can be treated with orthopedic surgery, the performance of skeletal and soft tissue surgical procedures may vary depending on the child’s GMFCS level [5-7]. After pelvic and femur osteotomies, children with CP sustain postoperative complications about twice as often as children who have similar hip surgery in, for example, developmental dysplasia of the hip or Légg–Calvé–Perthes disease [8]. Most studies on peri- and postoperative complications focus on specific types of surgery, such as scoliosis and reconstructive hip operations [9-13], but to our knowledge there are no population-based reports that describe the risks of complications after all types of elective orthopedic surgical interventions at all GMFCS levels. The aim was to assess the risk of complications after elective orthopedic surgery with respect to GMFCS level, anatomic location, and type of surgery (skeletal vs. soft tissue).

## Methods

### Design and setting

This study used population data from 3 national registers, the Swedish CP surveillance program – Cerebral Pares Uppföljnings Program (CPUP) [14], the National Patient Register (NPR) [15], and the National Cause of Death Register [16]. The STROBE guidelines were followed in the development and reporting of this study [17].

### Registers and data extracted

The NPR is maintained by the Swedish National Board of Health and Welfare. It contains information on all inpatient and outpatient healthcare contacts from all hospitals and outpatient clinics in Sweden [18]. Data from 1,536 individuals diagnosed with CP (G80) according to the International Statistical Classification of Diseases and Related Health Problems, 10th Revision (ICD-10) [19] were extracted from the NPR. Inpatient and outpatient data included information on year of birth, sex, period of hospital stay, diagnosis, and surgical procedure codes for orthopedic surgery. The data included hospital readmissions and outpatient contacts for up to 3 months after the end of the index hospital stay. Data on deaths was obtained from the National Cause of Death Register.

The CPUP surveillance program was initiated in southern Sweden in 1994 as a regional follow-up program for children with CP. From birth year 2001 almost all children with CP in Sweden are included, and since 2011 to include adults. More than 95% of children with CP in Sweden are followed up in the CPUP [2,20]. From the CPUP register, data on year of birth, sex, and GMFCS level were extracted for 1,536 individuals born between 1990 and 2019.

### Study cohort

**Figure 1:**
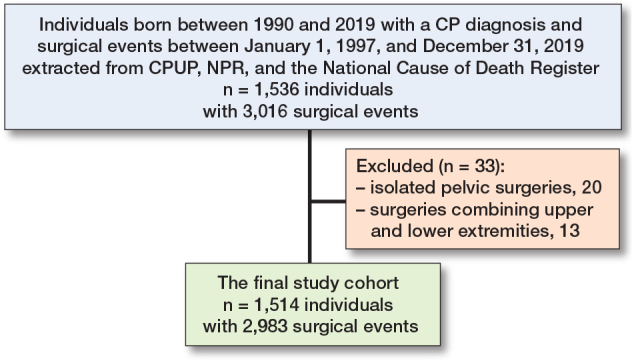
Patient flowchart.

The final study cohort consisted of all patients fulfilling the following criteria: followed up in CPUP and registered in NPR with elective orthopedic surgery between January 1, 1997, and December 31, 2019. Exclusion criteria were very rare orthopedic surgical events (Figure 1). The following parameters were analyzed: age, sex, GMFCS level, diagnosis codes including peri- and postoperative complications according to ICD-10, procedure codes for elective orthopedic surgical procedures, including anatomic level of surgery and assessment of type (skeletal or soft tissue).

### Classification of orthopedic surgeries

The orthopedic surgical procedure codes extracted from the NPR were based on the Swedish version of the NOMESCO Classification of Surgical Procedures (NCSP 96). One surgical event could include several surgical procedure codes. From the collected data, 3 of the coauthors (AT, JvH, PA) each with > 15 years of experience as pediatric orthopedic specialists, had consensus-building sessions to decide whether a procedure code should be classified as soft tissue or skeletal surgery. All surgical events indicating an elective musculoskeletal surgical procedure were included in the study. Elective removals of orthopedic implants were thus included among the surgical events studied.

### Classification of complications

Complications were defined as “A medical problem that occurs during a disease, or after a procedure or treatment” in accordance with the National Cancer Institute [21]. The occurrence of complications was assessed by analysis of diagnosis and surgical procedure codes, without access to the medical records. The coauthors (AT, JvH, PA) held consensus-building sessions to decide whether a specific diagnosis or procedure code should be classified as a complication. Complications were classified into 1 of 7 groups: infections, kidney and fluid balance, cardiopulmonary, gastroenterological, neurologic, skin, and miscellaneous. Severity of complications was graded into 3 groups: “life-threatening,” “potentially life-threatening,” or “non-life-threatening” (see Supplementary Data). These 3 groups were also collapsed into a fourth group: “any complication.” Complications that occurred during the hospital stay or within 3 months of discharge were included in the analysis. The cause of death could not be confirmed in this registry study as we did not have access to medical charts and therefore “death” was reported separately.

### Statistics

To characterize the sample, descriptive statistics were presented as counts, percentages, and means with ranges. The risk of complication in relation to GMFCS level, location of surgery, and type of surgery (i.e., skeletal or soft tissue) was analyzed by logistic regression. Odds ratios (ORs) with 95% confidence intervals (CIs) were estimated using the generalized estimating equation method to account for the potential dependence introduced by allowing multiple surgery admissions per patient. Furthermore, absolute complication risks were calculated for GMFCS levels I–III, IV, and V for each anatomic location and stratified according to type of surgery (i.e., skeletal vs. soft tissue). Two-tailed P values < 0.05 (corresponding to 95% CI of ORs not including unity) were used as a threshold for statistical significance. All statistical analyses were performed with SAS software using the GENMOD procedure (v. 9.4 SAS Institute, Inc, Cary, NC, USA).

### Ethics, funding, use of AI tools, and disclosures

Ethical approval was obtained from the Regional Ethical Review Board in Lund (DNR 2017/491, 2018/1000). Verbal consent to use the data for research was provided by all families participating in the CPUP. This work was supported by the following research funds: Stiftelsen Promobilia, Norrbacka-Eugeniastiftelsen, Stiftelsen för bistånd åt rörelsehindrade i Skåne, and Linnéa och Josef Carlssons Stiftelse. AI tools were not used in this study. The authors declare no conflict of interest. Complete disclosure of interest forms according to ICMJE are available on the article page, doi: 10.2340/17453674.2025.43705

## Results

### Patient characteristics

The cohort initially included 1,536 individuals with a CP diagnosis (G80) registered in NPR, and who were also being followed in CPUP (see Figure 1). As a first step in the analysis, the orthopedic surgical procedures were grouped according to anatomic localization. To create groups appropriately sized for comparisons, 33 of the rarest surgical events, consisting of 20 isolated pelvic surgeries and 13 surgeries combining upper and lower extremities (1.1% of the total), were excluded. We thus analyzed 2,983 surgical events for elective orthopedic surgery in 1,514 individuals with CP. The demographic characteristics, GMFCS level, anatomic level of surgical event, and type of surgery (e.g., skeletal or soft tissue) are presented in Tables 1 and 2. Children at GMFCS level V formed the largest group in terms of both individuals (n = 426, 28%) and surgical events (n = 1,090, 36%). Most were performed at the hip/femur level (29%), and the ankle/foot (20%). 220 individuals underwent spine surgery across 277 surgical events, representing 9% of the 2,983 cases.

**Table 1:** Demographic characteristics and distributions of GMFCS level. Values are presented as counts (%)

Variable	Patients n = 1,514	Surgical events n = 2,983
Birth year		
1997–2001	437 (29)	1,030 (34)
2002–2004	357 (24)	719 (24)
2005–2008	384 (25)	707 (24)
2009–2015	336 (22)	527 (18)
Female	656 (43)	1,311 (44)
GMFCS level		
I	403 (27)	586 (20)
II	200 (13)	345 (12)
III	167 (11)	290 (10)
IV	318 (21)	672 (22)
V	426 (28)	1,090 (36)

GMFCS = Gross Motor Function

Classification System.

**Table 2:** Anatomic level and type of surgery (skeletal or soft tissue). Values are counts (%)

Variable	Surgical events n = 2,983
Anatomic level	
Spine	277 (9.3)
Hip/femur	869 (29)
Pelvis/hip/femur	226 (7.6)
Knee/lower leg	144 (4.8)
Ankle/foot	599 (20)
Lower extremity	
2 levels	390 (13)
3 levels	142 (4.8)
Upper extremity	336 (11)
Type of surgery	
Soft tissue	1,219 (41)
Skeletal	1,764 (59)

**Figure 2:**
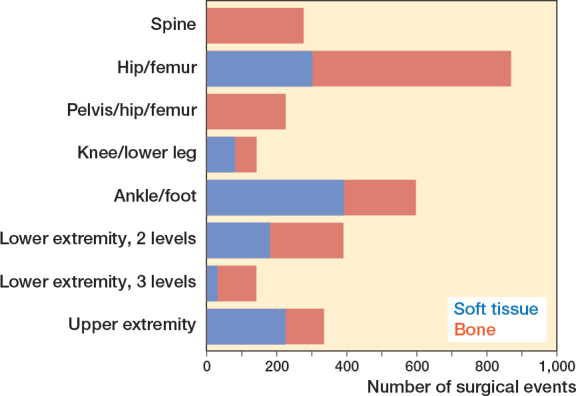
Distribution of type of surgery (soft tissue vs bone) according to anatomic level.

**Figure 3:**
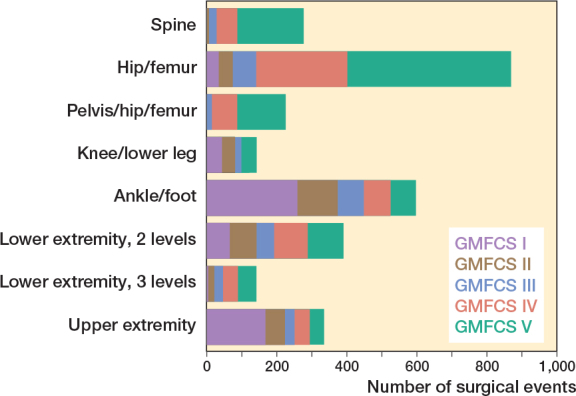
Absolute frequencies of operations with respect to the Gross Motor Function Classification System (GMFCS) level and anatomic level of surgery.

**Figure 4:**
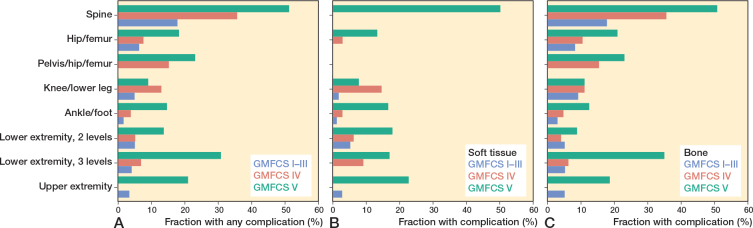
(A) Fraction of surgical events with complications in different Gross Motor Function Classification System (GMFCS) levels and anatomic level. (B) Fraction of soft tissue surgical events with complications. The soft tissue surgical event in the spine was a reoperation for a superficial infection. (C) Fraction of skeletal surgical events with complications. Note that GMFCS levels I–III are pooled together.

The proportions between skeletal and soft-tissue surgery varied considerably among anatomic levels, but the majority of surgical events included skeletal procedures (59%) (Figure 2). Register data did not contain consistent information on whether surgery was bilateral or one-sided. Therefore the standard classification of multilevel surgery (2 or more soft-tissue or bony surgical procedures on 2 or more anatomic levels during the same surgical session) could not be used [22]. Instead, surgical events involving more than 1 anatomic level of the lower extremity were classified according to whether 2 (e.g., hip/femur and foot/ankle) or 3 anatomic levels (e.g., hip/femur and knee/lower leg, and foot/ankle) were involved (Table 2 and Figures 2–4).

The distribution of orthopedic surgical events according to GMFCS level and anatomic level is shown in Figure 3. Spine, pelvic, and hip surgeries were most frequent at GMFCS levels IV and V, whereas foot/ankle and upper extremity surgical events were more common at GMFCS levels I and II. Surgical events involving 2 levels of the lower extremity were relatively evenly distributed among the GMFCS levels, while surgical events involving 3 levels of the lower extremity were most common at GMFCS level V, becoming gradually less common in the lower GMFCS levels.

### Outcomes

Using the broadest criterion, “any complication”, at least 1 event was reported in 380 out of 2,983 surgical events (13%). Similarly, the complication rate was 6% (73/1,219) for soft tissue and 17% (307/1,764) for skeletal surgical events. Of 517 reported complications, the majority were related to infections (51%). For example pneumonia (30%) was followed by miscellaneous (16%) and cardiovascular complications (12%) (see Supplementary data).

Generally, the highest rate of complication occurred at GMFCS level V and to a considerably lower extent at GMFCS levels I–III, even when pooled together (Figure 4A). Multiple complications in the same surgical event were also clearly more frequent at GMFCS levels IV and V (Table 3). For soft tissue surgery, at least 1 complication occurred in 2% (14/661) at GMFCS levels I–III, and 11% (59/558) at GMFCS levels IV–V (Figure 4B). The corresponding numbers for skeletal surgical events were 9% (48/559) at GMFCS levels I–III and 23% (273/1,205) at GMFCS levels IV–V (Figure 4C). Regarding anatomic level, spinal surgery showed the highest rate for at least 1 complication at 44% (123/277), followed by surgery to the pelvis/hip/femur at 19% (43/226) and 3 levels of the lower extremity surgery at 15% (21/142).

**Table 3:** Distribution of GMFCS level and number of complications per surgical event. Values are presented as counts (% per level)

GMFCS level	overall	Number of surgical events			
		without complication	with 1complication	with 2complications	with > 2complications
I	586	570 (97)	14 (2.4)	1 (0.2)	1 (0.2)
II	345	328 (95)	14 (4.1)	3 (0.9)	0 (0)
III	290	275 (95)	13 (4.5)	2 (0.7)	0 (0)
IV	672	606 (90)	55 (8.2)	10 (1.5)	1 (0.1)
V	1,090	824 (76)	177 (16)	63 (5.8)	26 (2.4)
Total	2,983	2,603 (87)	273 (9.2)	79 (2.6)	28 (0.9)

GMFCS: Gross Motor Function Classification System.

Regarding relative risk for “any complication,” ORs were statistically significantly higher in skeletal (1.6, CI 1.2–2.1) than in soft tissue surgical events (Table 4).

**Table 4:** Odds ratios (ORs) with 95% confidence intervals (CI) for “any complication” with respect to the Gross Motor Function Classification System (GMFCS) level, anatomic level of surgery, and type of surgery. ORso indicates odds ratio for soft tissue surgery and ORsk the odds ratio for skeletal surgery

Variable	OR (CI)	ORso (CI)	ORsk (CI)
GMFCS level			
I	1.0 (Ref.)	1.0 (Ref.)	1.0 (Ref.)
II	1.7 (0.7–3.9)	1.9 (0.5–7.2)	1.3 (0.5–3.7)
III	1.5 (0.6–3.5)	3.3 (0.9–11.8)	0.9 (0.3–2.4)
IV	2.5 (1.3–5.0)^a^	3.5 (1.1–10.9)^a^	1.7 (0.8–3.9)
V	7.0 (3.7–13.5)^a^	14.7 (5.4–40.1)^a^	4.0 (1.8–8.8)^a^
Anatomic level			
Ankle/foot	1.0 (Ref.)	1.0 (Ref.)	1.0 (Ref.)
Spine	7.9 (4.5–13.6)^a^	n.a.(	9.2 (4.5–9.0)^a^
Hip/femur	1.8 (1.1–3.0) ^a^	0.9 (0.4–1.9)	2.3 (1.1–4.6)^a^
Pelvis/hip/femur	2.3 (1.3–4.2)^a^	n.a.((	2.8 (1.3–5.9)^a^
Knee/lower leg	1.9 (0.8–4.2)	1.2 (0.4–4.1)	1.9 (0.5–6.6)
Lower extremity			
2 levels	1.6 (0.9–2.9)	1.6 (0.7–3.7)	1.2 (0.5–2.8)
3 levels	2.5 (1.3–4.8)^a^	1.3 (0.3–5.3)	2.9 (1.3–6.7)^a^
Upper extremity	1.8 (0.9–3.4)	1.6 (0.7–3.8)	1.5 (0.6–3.9)
Type of surgery			
Soft tissue	1.0 (Ref.)		
Bone	1.6 (1.2–2.1)^a^		

n.a. = not applicable; Ref. = Reference.

a Statistically significant.

Generally, ORs were statistically significantly increased at GMFCS level V and to a lesser extent at GMFCS level IV, also for soft tissue surgical events, as compared with GMFCS level I. In contrast, the ORs at GMFCS levels II and III were not statistically significantly different from children at GMFCS level I.

Regarding anatomic level, spinal surgery showed the highest OR, but also surgery involving the hip/femur region (i.e., hip/femur, pelvis/hip/femur, and 3 levels of the lower extremity) showed statistically significant increases in OR, except for soft tissue surgical events (see Table 4).

Sharpening the criterion for complications to “life-threatening or potentially life-threatening” resulted in statistically significantly increased OR at GMFCS levels V and IV as well as for spine and pelvis/hip/femur surgery, but not for skeletal vs. soft tissue surgery. 191 patients experienced “life-threatening or potentially life-threatening” events diagnosed in inpatient care, and 20 of these were categorized as “life-threatening” (Table 5).

**Table 5:** Odds ratios (ORs) with 95% confidence intervals (CI) for “life-threatening or potentially life-threatening” complications, with respect to the Gross Motor Function Classification System (GMFCS) level, anatomic level of surgery, and type of surgery

Variable	OR (CI)
GMFCS level	
I	1.0 (Ref.)
II	3.1 (0.9–11.4)
III	1.6 (0.3–7.5)
IV	5.1 (1.8–14.6) ^a^
V	13.5 (4.8–38.2) ^a^
Anatomic level	
Ankle/foot	1.0 (Ref.)
Spine	8.6 (4.4–16.9) ^a^
Hip/femur	1.1 (0.5–2.1)
Pelvis/hip/femur	2.6 (1.3–5.6) ^a^
Knee/lower leg	1.1 (0.3–3.8)
Lower extremity	
2 levels	1.0 (0.5–2.3)
3 levels	2.0 (0.9–4.6)
Upper extremity	1.7 (0.7–4.1)
Type of surgery	
Soft tissue	1.0 (Ref.)
Bone	1.0 (0.7–1.6)

a Statistically significant.

58 individuals had died by the end of the study period. 8 (6 boys) of these children died within 90 days after surgery. All of them were classified as GMFCS V and had been operated on with scoliosis surgery (n = 4), skeletal hip/femur surgery (n = 2), soft tissue hip surgery (n = 1), and soft tissue surgery of the upper limb (n = 1).

## Discussion

The aim of this study was to assess the risk of complications following elective orthopedic surgery in children and young adults with CP. The main study finding was that 13% of surgical events had at least 1 postoperative complication (6% in soft tissue surgeries, 17% in skeletal surgeries), and 51% of these were related to infection.

The rate of complications was higher among children with greater functional impairment, as indicated by higher GMFCS levels. The highest risk was observed after spinal surgery. There was no statistically significant difference in risk of complications (ORs) between GMFCS levels I, II, and III.

Statistically significantly increases in odds ratios (ORs) were found in more complex procedures such as skeletal surgery to the spine and hip/femur, which were almost exclusively performed in children at GMFCS levels IV–V. These elevated ORs align with earlier reports on respiratory complications, wound infections, and decubitus ulcers. Factors more common at GMFCS levels IV–V that have been reported to increase the risk of complications include the presence of a ventriculoperitoneal shunt, severe cognitive impairment, malnutrition, and the presence of a G-tube [23-25]. Gastrointestinal complications are also important to recognize after scoliosis surgery in children with CP. Verhofste et al. reported, in a study based on medical records of 425 children with CP surgically treated for scoliosis, that postoperative acute pancreatitis occurred in 45 cases (10.6%). Of these cases, 60% were classified as mild, while 40% were moderately severe and required GI-tube placement [26]. Although postoperative pancreatitis can be difficult to diagnose in asymptomatic patients, it is surprising that only 1 case was identified in this registry study, raising concerns about the potential for missing or misclassified diagnostic codes.

Skeletal surgery on the hip/femur region carried approximately twice the risk of complications compared with ankle/foot surgery. The ORs were further increased by including pelvic surgery, or by involving the knee/lower leg as well as the ankle/foot region. These complex lower extremity surgical events were also predominantly performed in the children at GMFCS levels IV–V (see Figure 3). As about 70% of the “3 levels of the lower extremity” surgical events were performed in wheelchair-bound children, they should not be considered as “single event multilevel surgeries” to improve gait. In contrast, soft tissue surgical events of “3 levels of the lower extremity” were not associated with an increased complication risk.

The high OR for soft tissue surgery at GMFCS level V (14.7, CI 5.4–40.1) is probably partly a consequence of the very low complication rate in the reference group, that is, soft tissue surgical events at GMFCS level I, and partly an effect of the more frequent comorbidities at GMFCS level V, as described earlier. Moreover, some children at GMFCS level V may be considered too weak for skeletal surgery, and judicious use of soft tissue surgery is occasionally used in selected cases.

A significantly higher overall risk of complications was identified in skeletal surgical procedures compared with soft tissue, likely due to pseudarthroses, malunion, complications with osteosynthesis materials and removal of them.

### Strengths

This is a large population-based cohort using data from 3 high-quality national registers, enhancing the generalizability of the findings.

The quality of the NPR has been systematically reviewed and the accuracy of the coding is reported to be high and the underreporting low [18]. In an earlier study, there was very high agreement in coverage between CPUP and the NPR regarding individuals with CP [7].

### Limitations

Because the register data did not contain information on the grade or course of a likely complication, the Clavien–Dindo–Sink classification could not be used in our study [27]. Instead, the analysis of complications was based on assessments of diagnosis and surgical procedure codes. Although the classification was not tested for reliability or repeatability, and complication rates are difficult to compare with other studies, the calculated ORs remain robust due to the large sample size and the comprehensive data collection methodology. Another limitation of registry data is the inability to analyze potential confounders, such as patient comorbidities and hospital-specific factors such as different hospital treatment regimens and the surgical volume that affects the experience of surgeons and the practice of postoperative care.

Missing data regarding bilateral surgical events, and also misclassification of diagnosis codes and surgical procedures that could not be validated in medical records, may affect complication rates and their interpretation. For example, the proportion of local infections could not be assessed.

### Conclusion

13% of all surgical events had at least 1 postoperative complication. Skeletal surgery is associated with a higher risk of postoperative complications compared with soft tissue orthopedic surgical procedures. Children classified as GMFCS level V are at an even higher risk, also after soft tissue surgery. Infections, particularly pneumonia, are the most frequently reported postoperative complications in this study.

*In perspective,* these findings provide valuable insights and are crucial to consider when deciding whether to proceed with an orthopedic surgical procedure. For children at GMFCS level V it is particularly important to monitor for complications both during hospitalization and after discharge.

### Supplementary data

A table with distribution of complications according to (ICD-10) is available as Supplementary data on the article page, doi: 10.2340/17453674.2025.43705

## Supplementary Material



## Notes

AT, JvH and PÅ wrote the first draft, which was revised by all authors. Study design and data collection: AT, JvH, FG, EB, GH, PÅ. Data analysis: AT, JvH, FG, GH, PÅ.

Handling co-editors: Ilkka Helenius

*Acta* thanks Matti Ahonen, Darko Anticevic, Freeman Miller, and Bjarne Møller-Madsen and other anonymous reviewers for help with peer review of this manuscript.

## References

[1] Himmelmann K. Putting prevention into practice for the benefit of children and young people with cerebral palsy. Arch Dis Child 2018; 103: 1100. doi: 10.1136/archdischild-2018-315134. 30021786

[2] Westbom L, Hägglund G, Nordmark E. Cerebral palsy in a total population of 4–11 year olds in southern Sweden: prevalence and distribution according to different CP classification systems. BMC Pediatr 2007; 7: 41. doi: 10.1186/1471-2431-7-41. 18053264 PMC2248184

[3] Hollung S J, Hägglund G, Gaston M S, Seid A K, Lydersen S, Alriksson-Schmidt A I, et al. Point prevalence and motor function of children and adolescents with cerebral palsy in Scandinavia and Scotland: a CP-North study. Dev Med Child Neurol 2021; 63(6): 721-8. doi: 10.1111/dmcn.14764. 33400264 PMC8247044

[4] Rosenbaum P L, Palisano R J, Bartlett D J, Galuppi B E, Russell D J. Development of the Gross Motor Function Classification System for cerebral palsy. Dev Med Child Neurol 2008; 50: 249-53. doi: 10.1111/j.1469-8749.2008.02045.x. 18318732

[5] Aversano M W, Sheikh Taha A M, Mundluru S, Otsuka N Y. What’s new in the orthopaedic treatment of cerebral palsy. J Pediatr Orthop 2017; 37: 210-6. doi: 10.1097/BPO.0000000000000675. 26523699

[6] Blumetti F C, Wu J C N, Barzi F, Axt M W, Waugh M C, Selber P. Orthopaedic surgery in dystonic cerebral palsy. J Pediatr Orthop 2019; 39: 209-16. doi: 10.1097/BPO.0000000000000919. 30839486

[7] Telléus A, Kiapekos N, Von Heideken J, Wagner P, Broström E, Hägglund G, et al. Orthopedic surgical procedures in 3,305 children and young adults with cerebral palsy: a register-based cohort study. Acta Orthop 2022; 93: 472-7. doi: 10.2340/17453674.2022.2583. 35611478 PMC9131193

[8] DiFazio R, Vessey J A, Miller P, Van Nostrand K, Snyder B. Postoperative complications after hip surgery in patients with cerebral palsy: a retrospective matched cohort study. J Pediatr Orthop 2016; 36: 56-62. doi: 10.1097/BPO.0000000000000404. 25633609

[9] Butler L R, Dominy C L, White C A, Mengsteab P, Lin E, Allen A K, et al. Risk factors for 90-day readmission and prolonged length of stay after hip surgery in children with cerebral palsy. J Orthop 2023; 38: 14-19. doi: 10.1016/j.jor.2023.03.002. 36925762 PMC10011680

[10] Yaszay B, Bartley C E, Sponseller P D, Abel M, Cahill P J, Shah S A, et al. Major complications following surgical correction of spine deformity in 257 patients with cerebral palsy. Spine Deform 2020; 8: 1305-1312. doi: 10.1007/s43390-020-00165-7. 32720268 PMC7384279

[11] Shea J, Nunally K D, Miller P E, Difazio R, Matheney T H, Snyder B, et al. Hip reconstruction in nonambulatory children with cerebral palsy: identifying risk factors associated with postoperative complications and prolonged length of stay. J Pediatr Orthop 2020; 40: e972-e977. doi: 10.1097/BPO.0000000000001643. 33045159

[12] Ruzbarsky J J, Beck NA, Baldwin K D, Sankar W N, Flynn J M, Spiegel D A. Risk factors and complications in hip reconstruction for nonambulatory patients with cerebral palsy. J Child Orthop 2013; 7: 487-500. doi: 10.1007/s11832-013-0536-1. 24432112 PMC3886352

[13] Lindén O, Lauge-Pedersen H, Hägglund G, Wagner P. Development of ankle and knee range of motion after isolated gastrocsoleus lengthening in children with cerebral palsy: a register-based longitudinal cohort study. Acta Orthop 2025; 96: 331-8. doi: 10.2340/17453674.2025.43387. 40242883 PMC12006035

[14] Cerebral Pares Uppföljnings Program (CPUP). Available from: https://www.cpup.se.

[15] National Patient Register (NPR). Available from: https://socialstyrelsen.se/en/statistics-and-data/registers/national-patient-register/.

[16] National Cause of Death Register. Available from: https://socialstyrelsen.se/en/statistics-and-data/registers/cause-of-death-register/.

[17] Vandenbroucke J P, von Elm E, Altman D G, Gøtzsche PC, Mulrow C D, Pocock S J, et al. Strengthening the Reporting of Observational Studies in Epidemiology (STROBE): explanation and elaboration. PLoS Med 2007; 4: e297. doi: 10.1371/journal.pmed.0040297. 17941715 PMC2020496

[18] Ludvigsson J F, Andersson E, Ekbom A, Feychting M, Kim J L, Reuterwall C, et al. External review and validation of the Swedish national inpatient register. BMC Public Health 2011; 11: 450. doi: 10.1186/1471-2458-11-450. 21658213 PMC3142234

[19] Park H, Castano J, Avila P, Perez D, Berinsky H, Gambarte L, et al. An Information retrieval approach to ICD-10 classification. Stud Health Technol Inform 2019; 264: 1564-5. doi: 10.3233/SHTI190536. 31438233

[20] Alriksson-Schmidt A I, Arner M, Westbom L, Krumlinde-Sundholm L, Nordmark E, Rodby-Bousquet E, et al. A combined surveillance program and quality register improves management of childhood disability. Disabil Rehabil 2017; 39: 830-6. doi: 10.3109/09638288.2016.1161843. 27044661

[21] National Cancer Institute. Available from: https://www.cancer.gov/publications/dictionaries/cancer-terms/def/complication.

[22] Harvey A, Graham H K, Morris M E, Baker R, Wolfe R. The Functional Mobility Scale: ability to detect change following single event multilevel surgery. Dev Med Child Neurol 2007; 49: 603-7. doi: 10.1111/j.1469-8749.2007.00603.x. 17635206

[23] Vandendriessche E, Proesmans M, Ortibus E, Moens P. Complication rate after scoliosis surgery in children with cerebral palsy. Acta Orthop Belg 2021; 87: 255-61. PMID: 34529378. 34529378

[24] Hasler C C. Operative treatment for spinal deformities in cerebral palsy. J Child Orthop 2013; 7: 419-23. doi: 10.1007/s11832-013-0517-4. 24432105 PMC3838513

[25] Nishnianidze T, Bayhan I A, Abousamra O, Sees J, Rogers K J, Dabney K W, et al. Factors predicting postoperative complications following spinal fusions in children with cerebral palsy scoliosis. Eur Spine J 2016; 25: 627-34. doi: 10.1007/s00586-015-4243-0. 26410446

[26] Verhofste B P, Berry J G, Miller P E, Crofton C N, Garrity B M, Fletcher N D, et al. Risk factors for gastrointestinal complications after spinal fusion in children with cerebral palsy. Spine Deform 2021; 9: 567-578. doi: 10.1007/s43390-020-00233-y. 33201495

[27] Dodwell E R, Pathy R, Widmann R F, Green D W, Scher D M, Blanco J S, et al. Reliability of the modified Clavien–Dindo–Sink complication classification system in pediatric orthopaedic surgery. JBJS Open Access 2018; 3: e0020. doi: 10.2106/JBJS.OA.18.00020. 30882054 PMC6400510

