# Supplementary material for: The risk of complications in elective orthopedic surgeries in children and young adults with cerebral palsy: a population-based register study
Source: Acta Orthop. 2025 May 27;96:387–93. doi: 10.2340/17453674.2025.43705 (PMC12212680; doi:10.2340/17453674.2025.43705)
Supplement: Supplementary file 1 [file ActaO-96-43705-s1.pdf]

Supplementary data

Supplementary Table. Classification and distribution of complications according to International Statistical Classification of Diseases and Related Health Problems, 10th Revision (ICD-10). The severity of complications is graded as life-threatening (L), potentially life-threatening (P), or non-life-threatening (N). Values are presented as counts (%)

ICD-10 code and specification		Events n = 517	Severity
**Infections**		**265 (51)**	
J18	Pneumonia, organism unspecified	101 (20)	P
J15	Bacterial pneumonia, not elsewhere classified	46 (9)	P
B96	Other bacterial agents as the cause of diseases classified to other chapters	24 (5)	N
B95	Streptococcus and staphylococcus as the cause of diseases classified to other chapters	23 (4)	N
A09	Diarrhea and gastroenteritis of presumed infectious origin	16 (3)	N
J20	Acute bronchitis	15 (3)	N
A08	Viral and other specified intestinal infections	8 (2)	N
A41	Other septicemia	7 (1)	P
M86	Osteomyelitis	6 (1)	N
A04	Other bacterial intestinal infections	5 (1)	P
A49	Bacterial infection of unspecified site	4 (1)	N
G04	Encephalitis, myelitis and encephalomyelitis	2 (<1)	P
J12	Viral pneumonia, not elsewhere classified	2 (<1)	P
J13	Pneumonia due to Streptococcus pneumoniae	2 (<1)	P
J14	Pneumonia due to Haemophilus influenzae	2 (<1)	P
G03	Meningitis due to other and unspecified causes	1 (<1)	N
G00	Bacterial meningitis, not elsewhere classified	1 (<1)	L
**Miscellaneous**		**82 (16)**	
T84	Complications of internal orthopedic prosthetic devices, implants and grafts	63 (12)	N
T88	Other complications of surgical and medical care, not elsewhere classified	13 (3)	P
T85	Complications of other internal prosthetic devices, implants and grafts	3 (1)	P
T79	Certain early complications of trauma, not elsewhere classified	2 (<1)	P
T80	Complications following infusion, transfusion and therapeutic injection	1 (<1)	P
**Fluid balance and kidney**		**74 (14)**	
D62	Acute posthemorrhagic anaemia	17 (3)	P
E87	Other disorders of fluid, electrolyte and acid-base balance	17 (3)	P
E86	Volume depletion	16 (3)	P

ICD-10 code and specification		Events n = 517	Severity
N10	Acute tubulointerstitial nephritis	13 (3)	P
R33	Retention of urine	6 (1)	N
N17	Acute renal failure	3 (1)	P
R57	Shock, not elsewhere classified	2 (<1)	L
**Cardiopulmonary**		**61 (12)**	
J96	Respiratory failure, not elsewhere classified	25 (5)	P
R09	Other symptoms and signs involving the circulatory and respiratory systems	9 (2)	P
J95	Postprocedural respiratory disorders, not elsewhere classified	6 (1)	P
I46	Cardiac arrest	6 (1)	L
I27	Other pulmonary heart diseases	3 (1)	P
J93	Pneumothorax	3 (1)	P
I74	Arterial embolism and thrombosis	3 (1)	L
R04	Hemorrhage from respiratory passages	2 (<1)	P
I50	Heart failure	1 (<1)	P
I82	Other venous embolism and thrombosis	1 (<1)	P
J86	Pyothorax	1 (<1)	P
I81	Portal vein thrombosis	1 (<1)	L
**Skin**		**27 (5)**	
L89	Decubitus ulcer	24 (5)	N
M96	Postprocedural musculoskeletal disorders, not elsewhere classified	2 (<1)	N
R02	Gangrene, not elsewhere classified	1 (<1)	L
**Gastro**		**4 (1)**	
K91	Postprocedural disorders of digestive system, not elsewhere classified	2 (<1)	N
K65	Peritonitis	1 (<1)	L
K85	Acute pancreatitis	1 (<1)	L
**Neuro**		**4 (1)**	
I63	Cerebral infarction	4 (1)	L

N39.0, Urinary tract infection site not specified (n = 50), was considered too unspecific and was therefore not classified as a complication.
